# Dipeptidyl Peptidase 4 Inhibition May Facilitate Healing of Chronic Foot Ulcers in Patients with Type 2 Diabetes

**DOI:** 10.1155/2012/892706

**Published:** 2012-11-01

**Authors:** Raffaele Marfella, Ferdinando Carlo Sasso, Maria Rosaria Rizzo, Pasquale Paolisso, Michelangela Barbieri, Vincenzo Padovano, Ornella Carbonara, Pasquale Gualdiero, Pasquale Petronella, Franca Ferraraccio, Antonello Petrella, Raffaele Canonico, Ferdinando Campitiello, Angela Della Corte, Giuseppe Paolisso, Silvestro Canonico

**Affiliations:** ^1^Department of Geriatrics and Metabolic Diseases, Second University of Naples, 80138 Naples, Italy; ^2^Department of Internal and Experimental Medicine, Center of Cardiovascular Excellence, Second University of Naples, 80138 Naples, Italy; ^3^Department of Geriatric Surgery, Second University of Naples, 80138 Naples, Italy; ^4^Department of Biochemistry, Section of Pathology, Second University Naples, 80138 Naples, Italy; ^5^Department of Pharmaceutical Sciences, University of Salerno, Salerno 84084, Italy

## Abstract

The pathophysiology of chronic diabetic ulcers is complex and still incompletely understood, both micro- and macroangiopathy strongly contribute to the development and delayed healing of diabetic wounds, through an impaired tissue feeding and response to ischemia. With adequate treatment, some ulcers may last only weeks; however, many ulcers are difficult to treat and may last months, in certain cases years; 19–35% of ulcers are reported as nonhealing. As no efficient therapy is available, it is a high priority to develop new strategies for treatment of this devastating complication. Because experimental and pathological studies suggest that incretin hormone glucagon-like peptide-1 may improves VEGF generation and promote the upregulation of HIF-1**α** through a reduction of oxidative stress, the study evaluated the effect of the augmentation of GLP-1, by inhibitors of the dipeptidyl peptidase-4, such as vildagliptin, on angiogenesis process and wound healing in diabetic chronic ulcers. Although elucidation of the pathophysiologic importance of these aspects awaits further confirmations, the present study evidences an additional aspect of how DPP-4 inhibition might contribute to improved ulcer outcome.

## 1. Introduction

The chronic foot ulcer is a leading cause of hospital admissions for people with diabetes in the developed world and is a major morbidity associated with diabetes, often leading to pain, suffering, and a poor quality of life for patients [1]. Its annual incidence is 2-3% and 7% in patients with neuropathy. Moreover, chronic diabetic foot ulcers are estimated to occur in 15% of all patients with diabetes and precede 84% of all diabetes-related lower-leg amputations [2, 3]. The pathophysiology of chronic diabetic foot ulcers is complex and still incompletely understood; both micro- and macroangiopathy as well as neuropathy strongly contribute to development and delayed healing of diabetic wounds, through an impaired tissue feeding and response to ischemia [4]. With adequate treatment, some ulcers may last only weeks; however, many ulcers are difficult to treat and may last months, in certain cases years, 19–35% of foot ulcers are reported as nonhealing [5, 6]. Therefore, it is a high priority to develop new strategies for treatment of this devastating complication [7, 8]. Important factors in the healing process include not only macrocirculation, but, more specifically, the local skin microcirculation and oxygenation of the tissue surrounding the ulcer. In this context, it has become increasingly evident that hypoxia plays an important role [9]. A critical stimulus for normal wound healing is relative hypoxia [10], and an impaired reaction to hypoxia could contribute to impaired wound healing. Local relative hypoxia in wounds was proved by direct measurement of the local oxygen pressure together with the necessity of maintaining hypoxic gradients for good angiogenesis in the wound healing process [10]. Adaptive responses of cells to hypoxia are mediated by the hypoxia-inducible factor-1 (HIF-1*α*), which is transcriptional factor that is expressed in response to a decrease in the partial pressure of cellular oxygen and activates the transcription of gene whose protein products mediate adaptive responses to hypoxia [11]. HIF-1*α* is an 826-amino acid protein that functions as a transacting transcriptional activator of vascular endothelial growth factor (VEGF) and inducible nitric oxide (NO) synthase (iNOS) [12]. Peak of expression of HIF-1*α* and VEGF, as well as the NO production from iNOS, may contribute to limitation of hypoxic injury by promoting angiogenesis and wound healing [12]. In essence, HIF-1*α* is necessary for expression of multiple angiogenic growth factors, cell motility, and recruitment of endothelial progenitor cells [12]. It has been shown that diabetes impairs HIF-1*α* and VEGF expressions [13, 14], as well as low levels of HIF-1*α* expression in foot ulcer biopsies in patients with diabetes, have been evidenced [15]. Finally, regulation of HIF activity is dependent on the oxidative stress activity and results in its degradation by the proteasome pathway [16]. Consequently, there is a need for more effective therapies that will address the physiological deficiencies that underlie the chronic ulcer. Because experimental and pathological studies suggest that incretin hormone glucagon-like peptide-1 (GLP-1) may improve VEGF generation, [17] and promote the upregulation of HIF-1*α* through a reduction of oxidative stress [18], the study evaluated the effect of the augmentation of GLP-1, by inhibitors of the dipeptidyl peptidase-4 (DPP-4), such as vildagliptin, on nitrotyrosine, proteasome 20S activity, HIF-1*α*, VEGF and capillary density, and wound healing in diabetic chronic ulcers.

## 2. Research Design and Methods

The study group comprised 106 type 2 diabetic patients with chronic nonhealing diabetic foot ulcers for more than 3-month duration. Patients were enrolled in the study between December 2008 and March 2011. Fifty-three diabetic patients were randomized (simple randomization, open labeled) to receive vildagliptin (50 mg b.i.d., *n* = 53) in addition to other concomitant hypoglycemic medication for 3 months. In the control group (*n* = 53), the dose of other concomitant hypoglycemic medication was changed to obtain a similar profile of metabolic parameters among the groups. Additional antidiabetic therapy, including sulfonylurea, metformin, and insulin, was titrated for optimal glycemic control for 3 months.

**Inclusion Criteria**

All patients had diabetes and at least one full-thickness wound below the ankle. Evaluations for both groups were made weekly until complete wound closure or the patient reached the week 12 visit without healing. All patients were assessed by a vascular surgeon at the time of inclusion and only patients with adequate distal perfusion were included in the study. Blood circulation (perfusion) was assessed by a dorsum transcutaneous oxygen test >30 mmHg, ankle-brachial index values >0.7 and <1.2 with toe pressure >30 mmHg, or Doppler arterial waveforms that were triphasic or biphasic at the ankle of the affected leg. Adequate distal perfusion was identified by fullfield circulatory parameters.

**Exclusion Criteria**

Patients with recognized active Charcot disease or ulcers resulting from electrical, chemical, or radiation burns and those with collagen vascular disease, ulcer malignancy, untreated osteomyelitis, or cellulitis were excluded from the study. Exclusion criteria also included ulcer treatment with normothermic or hyperbaric oxygen therapy; concomitant medications such as corticosteroids, immunosuppressive medications, or chemotherapy; recombinant or autologous growth factor products, skin and dermal substitutes within 30 days of study start, or use of any enzymatic debridement treatments. Pregnant or nursing mothers were excluded from study participation. Patients having an acute foot infection were included when the acute phase was resolved. Oral or local antibiotic treatment did not exclude patients from study participation.

**Study Treatments**

At each visit, tracings of the wound margins were made for computer planimetry to document changes in wound size, and photographs were taken for a visual record. At the screening visit, before randomization, and at each study visit study ulcers received sharp debridement and saline-moistened gauze dressings. The ulcers were debrided when considered necessary, and X-ray of the foot was performed in cases of suspected osteomyelitis. Individualized topical treatment and dressings were used depending on the site and character of the ulcer. Off-loading protective shoe wear with individually fitted insoles was used. Footwear and external pressure against the ulcer were inspected at each visit and corrected by an orthotist when required. Necrotic tissue and the excess granulation or overgranulation were debrided with a scalpel so that the wound bed was accurately assessed and facilitates healing.

**Procedures**

Outcomes were measured every week during the treatment period and then at three-month intervals. The regular treatment at the multidisciplinary diabetes foot clinic included treatment of infection, debridement, off-loading, and metabolic control according to high international standards [19]. Wound size was evaluated as previously described [20]. Wound closure was defined as “full epithelialization of the wound with the absence of drainage.” An ulcer was considered healed only after closure was confirmed at the next weekly visit. Fasting plasma glucose, insulin, and serum lipids were measured at each study visit. Written informed consent was obtained from all patients before each examination. The local ethics review committee approved the study.

### 2.1. End Points

The primary end point was healing of the index ulcer. The index ulcer was defined as the ulcer with the largest area and a duration of at least three months at the time of inclusion. An ulcer was considered healed when it was completely covered by epithelial regeneration and remained so until the next visit in the study. Secondary end points reported in this paper are as follows: a reduction in ulcer surface area over time; the improvements in angiogenic factors: HIF-1*α*, VEGF, and capillary density.

### 2.2. Biopsy of Ulcer

Biopsy was performed from the periphery of the ulcer before and after treatment. After the local anesthesia specimens were taken from the periphery of the ulcer. Biopsies were performed using Biopsy Punch 8 mm (Kai Medical), after skin anesthesia with Mepivacaine 1%. In all patients, the biopsy has been made on the margin of the lesion including the 50% of margin and the remaining skin lesion. Hemostasis was achieved by compression therapy. Biopsy sites were closed with polypropylene sutures.

### 2.3. Analysis of Specimens

Half of each biopsy specimen was fixed in formalin, sectioned to a thickness of 5 *μ*m, mounted on slides, and stained with hematoxylin and eosin. The mounted specimens were then examined for evidence of ischemia or kept for immunohistochemistry. A portion of the other half of the specimen was frozen in liquid nitrogen for the following Western blot analysis, and the remaining portion was prepared for RT-PCR.

### 2.4. Granulation Tissue Score

Amounts of granulation tissue in proportion to the wound area, wound depth, and wound area were each graded on a 0 to 4 scale: full of granulation tissue with epithelialization =0; granulation tissue >75% of wound area =1; granulation tissue >50–75% of wound area =2; granulation tissue 25–50% of wound area =3; granulation tissue <25% of wound area =4. The wound area and the amount of granulation tissue in proportion to the wound area were assessed using a transparency grid overlay to measure the surface area and counting the number of square centimeters within the wound perimeter.

#### 2.4.1. Western Blot Analysis

 HIF-1*α* and VEGF were determined by immunoblot analyses. Samples were lysed in a buffer containing 1% Nonidet P-40 for 30 min at 4°C. The lysates were then centrifuged for 10 min at 10,000 g at 4°C. After centrifugation, 20 *μ*g of each sample were loaded, electrophoresed in polyacrylamide gel, and electroblotted onto a nitrocellulose membrane. Primary antibodies to detect HIF-1*α* and VEGF were obtained from Santa Cruz Biotechnology (Santa Cruz, CA) and were utilized according to the manufacturer's instructions. Each Western blot was repeated at least three times. 

#### 2.4.2. Measurement of RNA (RT-PCR)

Each sample was analyzed for the presence of transcripts encoding human VEGF, and HIF-1*α* as already published [21]. Total RNA was extracted using RNAzol reagent (Biotecx Laboratories) according to the manufacturer's protocol. Levels of HIF-1*α* and VEGF were measured by RT-PCR amplification by using the following primer sequences: human VEGF_165_ 5′3′ = ATGAACTTTCTGTCTTGGGTG and 3′5′ = TCACCGCCTCGGCTTGTCACA; Human HIF-1*α* 5′3′ = CTGCTTGGTGCTGATTTGTGA and 3′5′ = TCCTGTACTGTCCTGTGGTGA.

#### 2.4.3. Immunohistochemical Staining

 Portions of the biopsy specimens were fixed in 10% formalin and prepared as 5 *μ*m-thick tissue sections on slides. The paraffin was then removed with a xylene substitute (Hemo-De; Fisher Scientific), and the sections were rehydrated with ethanol gradient washes. Tissue sections were quenched sequentially in 3% hydrogen peroxide in aqueous solution and blocked with PBS/6% nonfat dry milk (Biorad) for 1 h at room temperature. The sections of tissue from patients were incubated with mouse monoclonal anti-VEGF (10 *μ*g/mL; cat. no. Sc-7269; Santa Cruz), mouse monoclonal anti-HIF-1*α* (200 *μ*g/mL; cat. no. Sc-7269; Santa Cruz), and antiproteasome 20S (1 mg/mL; Dako). Tyrosine nitration, an index of the nitrosylation of proteins by peroxynitrite and/or reactive oxygen species (ROS), was determined by immunohistochemistry as previously described [14]. Sections were incubated overnight with anti-nitrotyrosine rabbit polyclonal antibody (1 : 500 in PBS, vol/vol). Sections were washed with PBS and incubated with secondary antibody. Specific labeling was detected with a biotin-conjugated goat anti-rabbit IgG and avidin-biotin peroxidase complex (DBA, Milan, Italy). Quantitative analysis for histology was as follows: we determined the VEGF, HIF-1*α*, and nitrotyrosine rich areas. Analysis of experiments was performed with a PC-based 24-bit color image-analysis system (IM500, Leica Microsystem AG) as previously described [22]. 

### 2.5. Biochemical Ulcer Tissue Assays

For the quantitative measurement of the proteasome 20S activity, a specific SDS-activation kit (Boston-Biochem) was used following company instructions; the most common assessment of 20S activity in vitro 256 is done by measuring the hydrolysis of the fluorogenic 257 peptidyl substrate Suc-Leu-Leu-Val-Tyr-AMC by the SDS-activated proteasome. This substrate is cleaved by the chymotryptic-like activity of the proteasome releasing free AMC (7-amino-4-methylcoumarin) which can be efficiently detected using a fluorimeter. For quantitative measurements of the proteasome, the fluorimeter may be calibrated by generating a standard curve using AMC. This calibration allows for the calculation of the exact specific activity of the 20S on each individual fluorimeter. Nitrotyrosine was assayed into tissue sample with a kit supplied by Hycult Biotech. Active GLP-1 was detected from wound tissue samples and analyzed using the Active GLP-1 Assay Kit (D.B.A., Italy).

### 2.6. Capillary Density Analysis

Capillary density was measured using immunohistochemistry with monoclonal antibody directed against CD31 (Abcam, Cambridge UK). Fifteen fields of three different sections from each tissue sample were randomly examined using a light microscope (400x) by two blinded observers. Slides were examined using a Leica microscope fixed with a Leica DC500 camera for digitizing images. Quantitative evaluation of the CD31 was performed by counting the number of positive blood vessels in the dermis of the section. After measuring the total area of the tissue sample (periphery of the ulcer) using the Leica imaging analysis system, the number of positive blood vessels per square millimeter was calculated. 

### 2.7. Statistical Analysis

Data are presented as mean ± SD or *n* (%). Student's *t* Test, Kruskal-Wallis Test, and Chi-square test were used to compare continuous and categorical variables, when indicated. Correlation between nitrotyrosine levels and proteasome 20S activity was tested. *P* < 0.05 was considered statistically significant. All calculations were performed using SPSS 12 (Sun Microsystem).

## 3. Results

### 3.1. Enrollment of the Patients and Effects of the DPP-4 Inhibition on Ulcer Healing

During the inclusion period, a total of 164 eligible participants were registered; 16 (10%) did not consent to participation, 42 (26%) were excluded according to the study protocol, and 106 (65%) underwent randomization. The two groups had similar characteristics at baseline (Table 1). In all patients, the mean ulcer duration was 124 ± 25 days, median was 128 days (25% = 99, 75% = 141), and the median ulcer area was 4.1 cm^2^ (25% = 3, 75% = 5). Percentages of patients treated for study ulcer infections at baseline were 30% (16 of 53) for vildagliptin group and 29% (15 of 53) for control group. Fasting plasma glucose levels between weeks 0 and 12 decreased similarly in control group (*P* < 0.05) and vildagliptin group (*P* < 0.05); HbA1c levels showed a trend to decrease in both groups (*P* < 0.05). In the control group, glimepiride was tested and its daily dose was adjusted in four patients to a range of 1–5 mg/day. Metformin was adjusted during the study in three patients; the daily dose range was 500–1,800 mg/day. Insulin was adjusted in nine patients. No patient in either group developed any clinical events, such as myocardial infarction and stroke, during the study. There was no difference between the groups in sharp debridement type as well as in number of sharp debridements. Moreover, There was no difference between the groups in the offloading strategies (Table 1). The number of patients who developed study ulcer-related adverse events (i.e., local wound infection, osteomyelitis, and cellulitis) was significantly lower in the vildagliptin patients (6 of 53, 11%) than in the control patients (16 of 53, 31%; *P* < 0.05). A significant difference in wound closure rate at week 12 was observed between the vildagliptin and control groups, complete healing of the index ulcer occurred in 16/53 (31%) in the vildagliptin group and 8/53 (15%) in the control group (*P* < 0.05). The percent of patients who underwent a surgical procedure involving the study ulcer was 8% (4 of 53) in the vildagliptin group and 15% (8 of 53) in the control group (*P* < 0.220). Osteomyelitis was the predominant reason for surgery in both groups. Surgical intervention for minor amputation was made in 1 patient from the vildagliptin group and 2 patients from the control group. We performed 90 biopsies in the vildagliptin group and 91 biopsies in the control group.

### 3.2. Effects of the DPP-4 Inhibition on Active GLP-1 in Wound Homogenate

There was no difference in GLP-1 in wound lysates among the groups at baseline (vildagliptin group: 1.7 ± 0.6 pg/mg; control group: 1.9 ± 0.8 pg/mg). After 12 weeks of active treatment (Table 2), a notable finding was that vildagliptin strongly increased levels of noncleaved (and thus active) GLP-1 in wound lysates (vildagliptin group: 4.9 ± 1.1 pg/mg; control group: 2.1 ± 0.6 pg/mg). Notably, total wound GLP-1 levels were up to twofold higher in vildagliptin group as compared to levels of active GLP-1 in control group.

### 3.3. Effects of the DPP-4 Inhibition on the Ulcer Granulation Score

Before treatment, no statistically significant difference was found between the granulation tissue score for the vildagliptin group and control group (3.2 ± 0.5 versus 3.3 ± 1.1, *P* > 0.05). After treatment, the score decreased (reflected as more amount of granulation tissue) statistically significantly in the vildagliptin group compared with the control group at week 12 (1.3 ± 0.9 versus 2.4 ± 1.2, *P* < 0.05).

### 3.4. Effects of the DPP-4 Inhibition on the Ulcer Oxidative Stress

**Figure 1:**
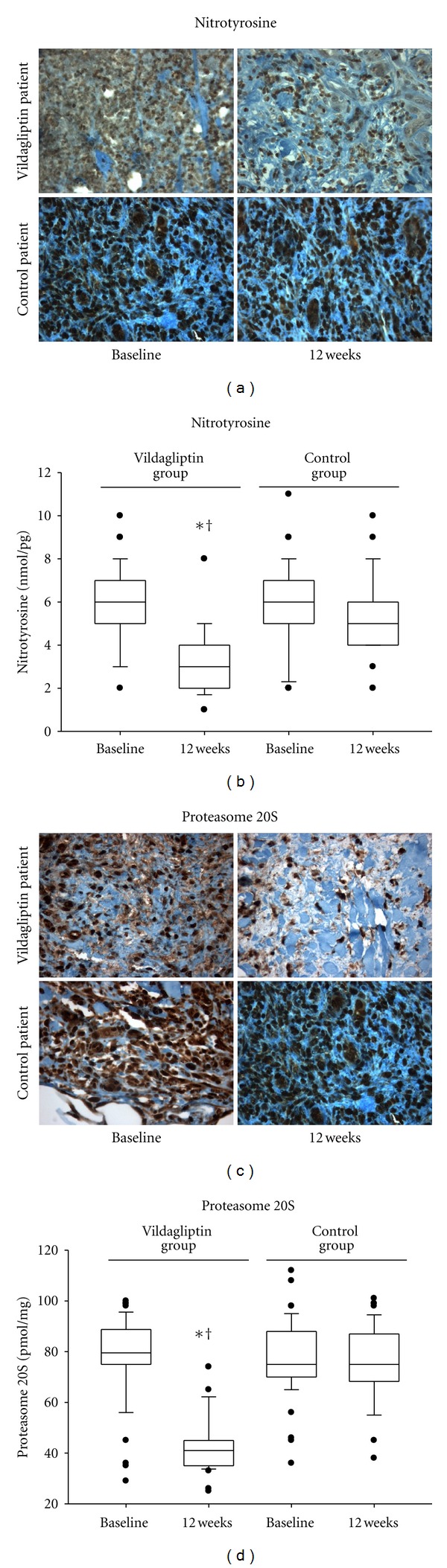
Effects of vildagliptin on nitrotyrosine levels and proteasome 20S activity in the periphery of the ulcer before and after treatment. Representative sections show immunochemistry for nitrotyrosine (a) (×400) and proteasome 20S (c) (×400) in ulcer specimens of control and vildagliptin patients at baseline and after 12 weeks of active treatment. Nitrotyrosine levels (b) and proteasome 20S activity (d) in ulcer specimens of control and vildagliptin patients at baseline and after 12 weeks of active treatment (boxplot, a plot type that displays the median; 10th, 20th, 25th, and 75th percentiles; range; extreme values). *P < 0.05 versus control group. ^†^ *P* < 0.05 versus baseline.

There was no difference in ulcer nitrotyrosine immunostaining between the groups at baseline (Figure 1). After 12 weeks of active treatment, specimen nitrotyrosine levels decreased significantly in the vildagliptin group (*P* < 0.01), while the nitrotyrosine level reductions did not reach significance in the control group (Figure 1). When immunostaining for the nitrotyrosine antigen was compared after 12 weeks of active treatment, a significant difference was found between tissues from vildagliptin and control groups. Higher levels, as well as higher staining, of nitrotyrosine were found in control compared with vildagliptin tissues from nonhealing wound patients (control, *n* = 45; vildagliptin group, *n* = 37) (*P* < 0.001) (Figure 1).

### 3.5. Effects of the DPP-4 Inhibition on the Ulcer Proteasome Activity

There was no difference in proteasome 20S immunostaining between the groups at baseline in ulcer tissues (Figure 1). Similarly, there was no difference in the proteasome activity between the groups at baseline in ulcer tissues (Figure 1). After 12 weeks of active treatments, strong immunostaining for proteasome 20S as well as high proteasome 20S activity was seen in biopsy from the periphery of the ulcer of tissues from nonhealing wound control patients (*n* = 45) (Figure 1). In contrast, immunostaining for proteasome 20S as well as proteasome 20S activity was almost undetectable in biopsy from the periphery of tissues from nonhealing wound vildagliptin patients (*n* = 37) (Figure 1). In vildagliptin patients, proteasome 20S activity was slightly detected in specimens from the periphery of the ulcer. Notably, proteasome activity in specimens from the periphery of the ulcer was strongly dependent on oxidative stress, as also reflected by the statistically significant correlation between nitrotyrosine levels and proteasome 20S activity (*R* = 0.42, *P* < 0.001).

### 3.6. Effects of the DPP-4 Inhibition on the Ulcer HIF-1*α* Expression

**Figure 2:**
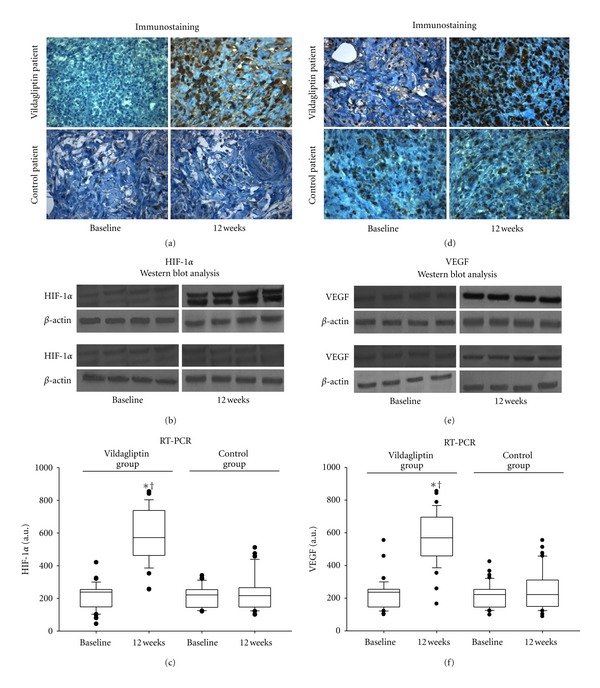
Effects of vildagliptin on HIF-1*α* and VEGF in the periphery of the ulcer before and after treatment. Representative sections show immunochemistry for HIF-1*α* (a) (×400) and proteasome 20S (d) (×400) in ulcer specimens of control and vildagliptin patients at baseline and after 12 weeks of active treatment. Western blot analysis of HIF-1*α* (b) and VEGF (e) content in ulcer specimens from control and vildagliptin patients. RT-PCR analysis of HIF-1*α* (c) and VEGF (f) content in ulcer specimens from control and vildagliptin patients. *P < 0.05 versus control patients. ^†^ *P* < 0.05 versus baseline.

Figure 2 shows the results of the analysis of HIF-1*α*, HIF-1*α* mRNA levels, and immunostaining from ulcer specimens from the groups of patients. The expression, mRNA levels, and immunostaining of HIF-1*α* in specimens from the periphery of the ulcer (*P* = 0.14) did not differ significantly between control and vildagliptin patients (Figure 2). After 12 weeks of active treatments, strong immunostaining, high expression, and mRNA levels of HIF-1*α* were seen in biopsy from the periphery of tissues in nonhealing wound vildagliptin patients (*n* = 37). In contrast, immunostaining, expression, and mRNA levels of HIF-1*α* were almost undetectable in biopsy from the periphery of tissues from nonhealing wound control patients (*n* = 37). In control patients, HIF-1*α* mRNA was slightly detected in specimens from the periphery of the ulcer; the values of HIF-1*α* increments were only 7% of the increments seen in biopsy of vildagliptin patients (*P* < 0.001). Notably, HIF-1*α* mRNA levels in specimens from the periphery of the ulcer were strongly dependent on proteasome activity, as also reflected by the statistically significant correlation between HIF-1*α* levels and proteasome 20S activity (*R* = 0.37,   *P* < 0.01).

### 3.7. Effects of the DPP-4 Inhibition on the Ulcer VEGF Expression

Figure 2 shows the results of the analysis of VEGF expression, mRNA levels, and immunostaining from ulcer specimens from the groups of patients. The expression, mRNA levels, and immunostaining of VEGF, in specimens from the periphery of the ulcer, did not differ significantly between control and vildagliptin patients (Figure 2). After 12 weeks of active treatments, strong immunostaining, high expression, and mRNA levels for VEGF were seen in biopsy from the periphery of tissues from nonhealing wound vildagliptin patients (*n* = 37). In contrast, immunostaining, expression, and mRNA levels of VEGF were almost undetectable in biopsy from the periphery of tissues from nonhealing wound control patients (*n* = 37). In control patients, VEGF mRNA levels were slightly detected in specimens from the periphery of the ulcer; the values of VEGF mRNA increments were only 8% of the increments seen in biopsy of vildagliptin patients (*P* < 0.001). 

### 3.8. Effects of the DPP-4 Inhibition on the Ulcer Capillary Density

**Figure 3:**
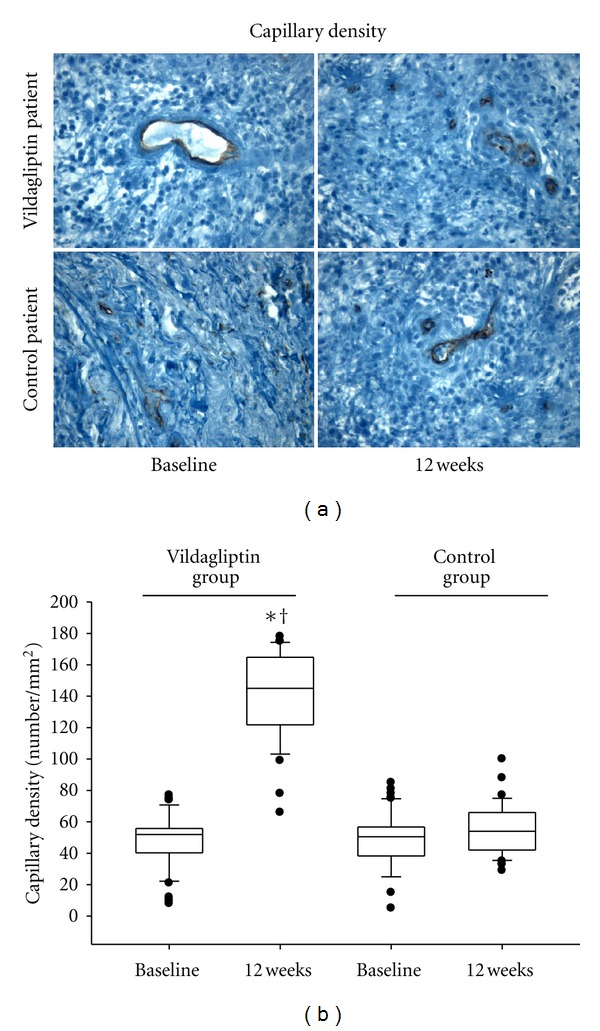
Effects of vildagliptin on capillary density in the periphery of the ulcer before and after treatment. (a) Representative images of the immunohistochemical staining with an anti-CD31 monoclonal antibody in the groups at ×400 magnification. (b) Graphic representation of CD31 positive cells expressed as the number of mm^2^. Data are presented as means ± DS. *P < 0.05 versus control group. ^†^ *P* < 0.05 versus baseline.

At baseline, the capillary density in the periphery of tissues from nonhealing wound patients (expressed as the number of capillaries per mm^2^) did not differ significantly between control and vildagliptin patients (Figure 3). After 12 months of active treatment, the qualitative differences between the control and vildagliptin specimens are shown in Figure 3(a); specimen capillary density levels increased significantly in the vildagliptin group (*P* < 0.01), while the capillary increments did not reach significance in the control group (Figure 3(a)). These differences were confirmed by qualitative image analysis of the sections. CD31 immunoreactivity was more prominent in vildagliptin than in control specimens (Figure 3).

## 4. Discussion

We have showed that DPP-4 inhibition accelerates healing of diabetic chronic ulcer, through induction of a histologic pattern consistent with enhanced angiogenesis. To the best of our knowledge, there has been no study evaluating the vildagliptin effect on the healing of foot ulcers in humans. In our study the main result has been represented by the significant difference in wound closure rate in diabetic patients treated with vildagliptin compared to diabetic control group. Indeed, at the same level of blood glucose levels, a significantly greater number of diabetic patients, treated with DPP-4 inhibitors, achieved complete ulcer closure than control diabetic patients. Moreover, we have observed an increase of HIF-1*α*, VEGF, capillary density and a decrease of nitrotyrosine levels in the specimens obtained in ulcers of the vildagliptin group both compared to their baseline and control group. Therefore, the results of the current study suggest that vildagliptin may have the ability to improve wound healing by increasing angiogenesis in the wound environment, normalizing the rate of oxidative stress, and making positive changes to capillary density. In diabetic patients treated with vildagliptin, we detected increased steady-state levels of HIF-1*α* and VEGF in specimens from ulcers. In control diabetic patients, the picture is quite different because both HIF-1*α* and VEGF levels were significantly lower than those in vildagliptin-treated ulcer specimens. HIF-1*α* plays a pivotal role in wound healing, and its expression in the multistage process of normal wound healing has been well characterized [11]. In essence, HIF-1*α* is necessary for expression of cell motility, and recruitment of endothelial progenitor cells and multiple angiogenic growth factors such as VEGF [23]. VEGF has an important role in stimulating the growth of new capillaries in several organ systems [24] and thus is a good candidate for the role of stimulating neovascularization to accelerate healing of diabetic chronic ulcer. We therefore hypothesized that the ability of DPP-4 inhibitors in wound healing is a result of an increase of HIF-1*α* and VEGF activity as well as of a reduction of oxidative stress. There are no studies in the literature that evaluated the effects of DPP-4 inhibitors on both HIF-1*α* and VEGF expressions in chronic ulcers. However, previous studies evidenced that GLP-1 improves VEGF generation in human pancreatic islet endothelial cells [17]. In this context, the mechanisms by which the vildagliptin may improve angiogenesis process in diabetic chronic foot ulcers remain poorly understood. Therefore, it is of importance to understand the mechanism of HIF-1*α*/VEGF pathway regulation after its induction in diabetic chronic ulcers. It is known that under normoxic conditions, HIF-1*α* is rapidly degraded via the ubiquitin dependent proteasomal 26S pathway after hydroxylation and ubiquitination. Under hypoxic conditions, it is generally regarded that HIF-1*α* is accumulated due to hydroxylation inhibition [25]. However, recent evidence has revealed that oxidative stress is involved in HIF-1*α* degradation in hypoxic conditions [26]. Although it is not clear how oxidative stress accelerates HIF-1*α* degradation in ischemic tissue, oxidative stress may promote HIF-1*α* degradation through the proteasomal degradation pathways. The proteasomal proteolytic pathways are among the principle mechanisms by which many intracellular proteins are degraded [27]. These pathways are critical in regulating important cell processes including differentiation, cytokine-induced gene expression, apoptosis, and the stress response [28]. The proteasome complexes exist in two forms, 26S and 20S. The two different complexes usually have different protein substrate specificities. The 26S proteasomal pathway is ubiquitin dependent and is a major pathway for protein degradation in cells. The 20S proteasomal pathway is ubiquitin independent and primarily degrades cellular oxidized proteins under conditions of oxidative stress [29]. Thus, oxidative stress may elevate 20S proteasomal activity [30]. Accordingly, in ulcer tissues from diabetic patients, we evidenced that nitrotyrosine levels were significantly correlated with proteasome activity. Thus, we can speculate that increased proteasome activity, as a consequence of oxidative stress overexpression, may enhance the degradation of HIF-1*α*, possibly representing a crucial step in the pathophysiology of impaired wound healing in the diabetic patients. Because of the study design, we cannot exclude whether the proteasome pathway exerts also a protective compensatory response, or whether it is merely a correlative marker for the reduced HIF-1*α* levels in diabetic lesions. However, the significant correlation between proteasome activity and the reduction of HIF-1*α* levels in ulcer tissues suggests an involvement of the proteasome system in the impaired wound healing of diabetic lesion by decreasing angiogenesis process. The present findings also show an inhibitory effect of DPP-4 inhibitors on proteasome activity in diabetic lesions. Indeed, at the same level of blood glucose levels, diabetic patients treated with vildagliptin had the lowest level of proteasome 20S activity, associated with the highest capillary density in ulcer tissues. Thus, patients assigned to vildagliptin had greater angiogenesis than patients treated without DPP-4 inhibitors. In particular, the reduced proteasome activity seen in diabetic ulcers of the vildagliptin group suggests decreased HIF-1*α* degradation and hence increased VEGF activation and increased capillary density. Our findings may have clinical implications because in a large series of diabetic ulcer specimens, it has been shown that angiogenesis is one of the major determinants of wound healing [31]. Previous data, in experimental models, suggest that both incretin mimetic agents and DPP-4 inhibitors agonists suggest a potentially beneficial role in diabetes-affected wound healing. [32, 33]. However, these studies did not provide any evidence about the effects of incretin on angiogenesis process or assess the specific pathway transducing environmental stimuli in enhanced wound healing of diabetic ulcers treated with DPP-4 inhibitors. In our study, the most straightforward interpretation is that DPP-4 inhibition raised GLP-1 concentrations, which reduced the activation of proteasome activity by oxidative stress. In this context, the decreased HIF-1*α* degradation and hence increased VEGF activation may promote the angiogenesis process and thus improve wound healing. An alternative explanation is that glucose lowering per se improved wound healing. However, the persistence of impaired wound healing in patients treated without DPP-4 inhibition, despite the decrease in blood glucose levels at study end, seem to argue against this hypothesis. In conclusion, the present study suggests an additional aspect of how DPP-4 inhibition might contribute to healing of chronic foot ulcers in patients with type 2 diabetes. 

### 4.1. Study Limitations

 The major limitation of our study is that this is an open-labeled study. Therefore, this ancillary effect of vildagliptin might have favorable implications in diabetes complications but needs to be confirmed in larger and longer outcome studies as well as in double blind clinical trials. Finally, we do not have the certainty that the assessments made on our biopsies definitively clarify the process of wound healing in diabetic patients. However, we hypothesize that the modulation of angiogenesis may play a role in the ulcer healing process.

## Figures and Tables

**Table 1:** Baseline characteristics of study patients.

	Control group	Vildagliptin group	*P *
*N *	53	53	
Mean age (years)	63 ± 15	64 ± 17	NS
Sex (M/F)	34/19	35/18	NS
BMI (kg/m^2^)	29 ± 2.8	30 ± 2.1	NS
Diabetes duration (years)	16 ± 6	17 ± 5	NS
Waist-to-hip ratio	0.91 ± 0.06	0.92 ± 0.09	NS
Systolic blood pressure (mmHg)	131 ± 18	129 ± 16	NS
Diastolic blood pressure (mmHg)	79 ± 8	80 ± 7	NS
Heart rate (bpm)	83 ± 26	84 ± 24	NS
Laboratory results			
Glycated hemoglobin (%)	8.0 ± 1.2	8.1 ± 1.3	NS
Plasma glucose (mg/dL)	168 ± 33	170 ± 36	NS
Creatinine (mg/dL)	1.1 ± 0.6	1.0 ± 0.8	NS
Colesterol (mg/dL)	230 ± 49	234 ± 51	NS
Triglycerides (mg/dL)	166 ± 63	171 ± 69	NS
HDL (mg/dL)	36 ± 12	35 ± 11	NS
LDL (mg/dL)	162 ± 23	165 ± 30	NS
Risk factors			
Hypertension (%)	32 (61)	33 (62)	NS
Hyperlipidemia (%)	25 (47)	26 (48)	NS
CAD (%)	22 (41)	21 (39)	NS
Stroke (%)	5 (10)	4 (7)	NS
Previous smokers (%)	19 (36)	20 (38)	NS
Active smokers (%)	5 (10)	6 (12)	NS
Therapy			
Insulin (%)	14 (26)	14 (26)	NS
Metformin (%)	26 (50)	28 (52)	NS
Sulfonylurea (%)	11 (21)	10 (19)	NS
Statin (%)	42 (80)	43 (81)	NS
Aspirin (%)	34 (64)	36 (67)	NS
Clopidogrel (%)	6 (11)	7 (13)	NS
Warfarin (%)	4 (7 )	5 (10)	NS
ACE-inhibitor or ARB (%)	31 (60)	30 (58)	NS
*β*-blocker (%)	14 (26)	15 (29)	NS
Diuretics (%)	33 (62)	36 (67)	NS
Ankle-brachial index (mmHg)	1.0 ± 0.1	1.0 ± 0.2	NS
Transcutaneous oxygen tension (mmHg)	44.9 ± 12.1	44.2 ± 11.8	NS
Ulcer duration before treatment (days)	122 ± 22	126 ± 26	NS
Plantar foot ulcers (%)	32 (61)	33 (62)	NS
Dorsal foot ulcers (%)	11 (21)	10 (19)	NS
Lateral foot ulcers (%)	10 (19)	10 (19)	NS
Baseline wound area (cm^2^)	4.1 ± 1.2	4.3 ± 1.5	NS
Off-loading methods			
Accommodative dressing (%)	30 (60)	30 (60)	NS
Healing shoe (%)	3 (6)	5 (9)	NS
Walking splint (%)	1 (2)	1 (2)	NS

Data are means ± SD or *n* (%).

**Table 2:** Clinical and metabolic parameters before and 3 months after active treatment.

	Control group			Vildagliptin group		
	Baseline	After 3 months	*P *	Baseline	After 3 months	*P *
	
BMI (kg/m^2^)	29.2 ± 2.8	28.3 ± 1.6	NS	30.1 ± 2.1	28.7 ± 1.2	NS
Systolic blood pressure (mmHg)	131 ± 18	130 ± 22	NS	129 ± 16	128 ± 20	NS
Diastolic blood pressure (mmHg)	79 ± 8	79 ± 10	NS	80 ± 7	78 ± 9	NS
Heart rate (bpm)	83 ± 26	81 ± 18	NS	84 ± 24	83 ± 21	NS
Laboratory results						
Glycated hemoglobin (%)	8.0 ± 1.2	7.1 ± 1.2	<0.05	8.1 ± 1.3	7.0 ± 1.0	<0.05
Plasma glucose (mg/dL)	168 ± 23	126 ± 15	<0.05	170 ± 36	131 ± 12	<0.05
Creatinine (mg/dL)	1.1 ± 0.6	1.05 ± 0.2	NS	1.0 ± 0.8	1.0 ± 0.5	NS
Cholesterol (mg/dL)	230 ± 49	228 ± 33	NS	234 ± 51	233 ± 29	NS
Tryglicerides (mg/dL)	166 ± 63	155 ± 45	NS	171 ± 69	159 ± 44	NS
HDL (mg/dL)	36 ± 12	38 ± 9	NS	35 ± 11	39 ± 12	NS
LDL (mg/dL)	162 ± 23	159 ± 22	NS	165 ± 30	162 ± 26	NS
Transcutaneous oxygen tension (mmHg)	44.9 ± 12.1	45.8 ± 13.8	NS	44.2 ± 11.8	46.3 ± 13.2	NS
Wound area (cm^2^)	4.1 ± 1.2	3.6 ± 2.1	NS	4.3 ± 1.5	1.2 ± 0.8*	<0.01

Data are means ± SD or *n* (%). *P < 0.05 compared to control group.
